# SCPS: a fast implementation of a spectral method for detecting protein families on a genome-wide scale

**DOI:** 10.1186/1471-2105-11-120

**Published:** 2010-03-09

**Authors:** Tamás Nepusz, Rajkumar Sasidharan, Alberto Paccanaro

**Affiliations:** 1 Centre for Systems and Synthetic Biology, Department of Computer Science, Royal Holloway, University of London, TW20 0EX, Egham, UK; 2 Department of Plant Biology, Carnegie Institution for Science, 260 Panama Street, Stanford, CA 94305, USA

## Abstract

**Background:**

An important problem in genomics is the automatic inference of groups of homologous proteins from pairwise sequence similarities. Several approaches have been proposed for this task which are "local" in the sense that they assign a protein to a cluster based only on the distances between that protein and the other proteins in the set. It was shown recently that global methods such as spectral clustering have better performance on a wide variety of datasets. However, currently available implementations of spectral clustering methods mostly consist of a few loosely coupled Matlab scripts that assume a fair amount of familiarity with Matlab programming and hence they are inaccessible for large parts of the research community.

**Results:**

SCPS (Spectral Clustering of Protein Sequences) is an efficient and user-friendly implementation of a spectral method for inferring protein families. The method uses only pairwise sequence similarities, and is therefore practical when only sequence information is available. SCPS was tested on difficult sets of proteins whose relationships were extracted from the SCOP database, and its results were extensively compared with those obtained using other popular protein clustering algorithms such as TribeMCL, hierarchical clustering and connected component analysis. We show that SCPS is able to identify many of the family/superfamily relationships correctly and that the quality of the obtained clusters as indicated by their F-scores is consistently better than all the other methods we compared it with. We also demonstrate the scalability of SCPS by clustering the entire SCOP database (14,183 sequences) and the complete genome of the yeast *Saccharomyces cerevisiae *(6,690 sequences).

**Conclusions:**

Besides the spectral method, SCPS also implements connected component analysis and hierarchical clustering, it integrates TribeMCL, it provides different cluster quality tools, it can extract human-readable protein descriptions using GI numbers from NCBI, it interfaces with external tools such as BLAST and Cytoscape, and it can produce publication-quality graphical representations of the clusters obtained, thus constituting a comprehensive and effective tool for practical research in computational biology. Source code and precompiled executables for Windows, Linux and Mac OS X are freely available at http://www.paccanarolab.org/software/scps.

## Background

An important problem in genomics is the automatic inference of groups of homologous proteins when only sequence information is available. Several approaches have been proposed for this task which are "local" in the sense that they assign a protein to a cluster based only on the distances between that protein and the other proteins in the set. In fact, the majority of these methods are based on thresholding a sequence similarity measure (e.g., BLAST E-value [1] or percent identity) and considering two protein sequences potentially homologous if their similarity is above the threshold [2,3]. However, by considering SCOP superfamilies as gold standard collections of homologous proteins and analysing the distribution of sequence distances within and between superfamilies, it was shown that there does not exist a single threshold on BLAST E-values that can be used to cluster homologues correctly [4]. As a consequence, while the existing methods yield adequate results for close homologues, they are likely to fail in identifying distant evolutionary relationships.

A possible way to improve these results is to use "global" methods, which cluster a set of proteins taking into account all the distances between every pair of proteins in the set. Paccanaro et al [4] introduced a global method based on spectral clustering and showed that it has better performance than commonly used local methods (namely hierarchical clustering [5] and connected component analysis [6]) and TribeMCL [7]. Other authors have also used spectral clustering successfully in various biological contexts [8-12]. The development of SCPS (Spectral Clustering of Protein Sequences) was motivated by the fact that currently available implementations of spectral clustering methods mostly consist of a few loosely coupled Matlab scripts that assume a fair amount of familiarity with Matlab programming and hence they are inaccessible for large parts of the research community. Moreover, the mathematical formulation of the algorithm is rather involved and it is not trivial to implement all the details properly in an ex-novo implementation.

SCPS provides an implementation of the spectral clustering algorithm [4] via a simple, clean and user-friendly graphical user interface that requires no background knowledge in programming or in the details of spectral clustering algorithms. SCPS is also able to perform connected component analysis and hierarchical clustering, and it incorporates TribeMCL, thus providing the user with an integrated environment where one can experiment with different clustering techniques. SCPS is extremely efficient and its speed scales well with the size of the dataset, allowing for the clustering of protein sets constituted by thousands of proteins in a few minutes. Moreover, SCPS is able to calculate different cluster quality scores, it interfaces with external tools such as BLAST [1] and Cytoscape [13], and it can produce publication-quality graphical representations of the clusters obtained, thus constituting a comprehensive tool for practical research. For more advanced use-cases (i.e., the integration of SCPS in automated batch processing pipelines), we also included a sophisticated command line interface.

SCPS was written in C++ and is distributed as an open-source package. Precompiled executables are available for the three major operating systems (Windows, Linux and Mac OS X) at http://www.paccanarolab.org/software/scps.

In the rest of this paper, we outline the general framework of our spectral clustering algorithm and then demonstrate its practical usage and usefulness via a number of benchmarks ranging from a few superfamilies to the entire SCOP database and the genome of the yeast *Saccharomyces cerevisiae*.

## Implementation

### Spectral clustering in SCPS

The goal of SCPS is to infer homology relations between protein sequences based on pairwise sequence information only. The input dataset thus consists of either a set of protein sequences or a list of pairwise similarity scores between some protein domains. The output is a partition of the sequences such that each sequence is assigned to one and only one of the partitions in a way that the partitions represent groups of homologs.

A typical SCPS workflow starts with either a FASTA file containing sequences for the protein domains of interest, or a list of BLAST E-values for all pairs of proteins where significant sequence similarity was reported by BLAST. Besides spectral clustering [4], SCPS currently supports connected component analysis, hierarchical clustering and TribeMCL [7], and more algorithms will be added in the near future. The spectral clustering approach reformulates the problem of protein homology detection into that of finding an optimal partition of a weighted undirected graph *G*. Each vertex of the graph corresponds to a protein sequence. Vertices are connected by undirected, weighted edges, each edge denoting a similarity relation between the two proteins it connects. The weight (label) of the edge is related to the probability of evolutionary relatedness. Edges with large weight are more likely to appear between domains of the same superfamily, hence the problem of partitioning the graph into subsets of vertices with mostly heavy-weight edges is an equivalent formulation of the original protein sequence classification problem. Spectral clustering solves the problem of finding the optimal partition by examining random walks on the similarity graph [14].

**Figure 1:**
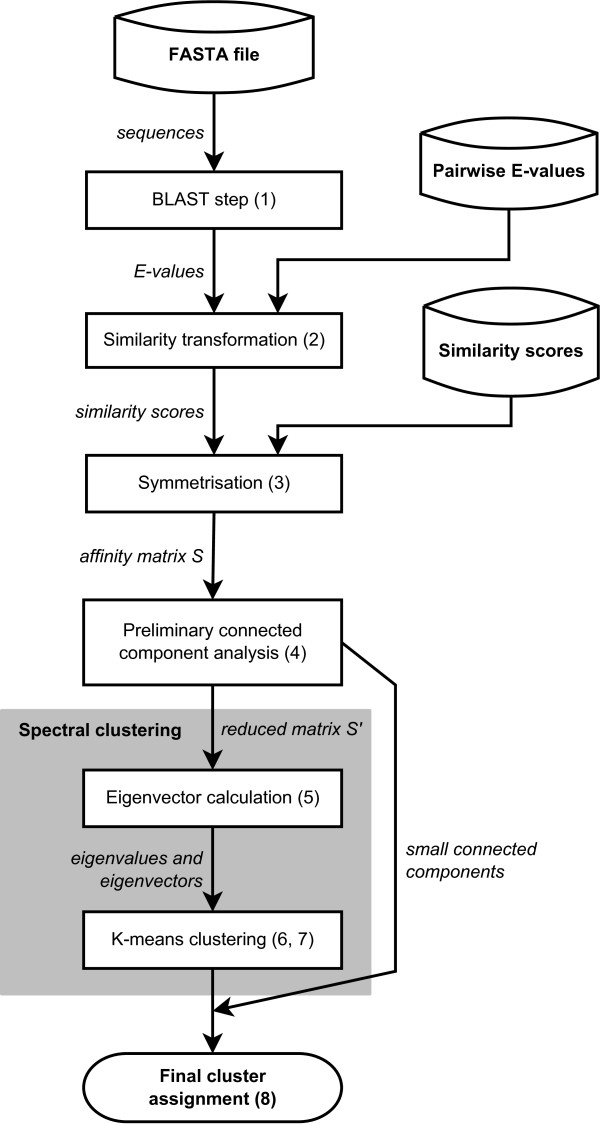
**The steps of the spectral clustering algorithm**. The main steps of the spectral clustering algorithm. Cylinders represent possible input file types, boxes represent processing steps. The numbers in the boxes refer to the steps of the algorithm as described in the main text.

Our approach is based on the spectral clustering algorithm of [15]. The general workflow is depicted on Figure 1. The basic steps of the algorithm are as follows:

1. If the input file is a FASTA sequence file, we conduct an all-against-all matching using BLAST and store the E-values.

2. Given the pairwise BLAST E-values obtained either from the previous step or directly from the input file, we build an affinity matrix based on a non-linear transformation from E-values to similarity scores. The matrix element in row *i *and column *j *contains the E-value corresponding to protein *j *when protein *i *was used as a query sequence.

3. Since the BLAST E-value corresponding to a query protein *i *matching protein *j *in the database is not necessarily equal to the case when the query protein *j *matches protein *i *in the same database, the affinity matrix has to be symmetrized. To obtain a symmetric matrix, we take the higher similarity score (i.e. the smaller E-value) in case of ambiguity. Let *s*_*ij *_denote the symmetrized similarity score between protein *i *and protein *j*. The *s*_*ij *_values together constitute the symmetrized affinity matrix **S**, whose main diagonal contains only ones.

4. We conduct a preliminary connected component analysis on the graph represented by the affinity matrix **S **to identify small connected components containing less than five sequences. It is unlikely that these components should be subdivided further, therefore we remove the rows and columns corresponding to these sequences from **S**, obtaining a reduced matrix **S**^'^.

5. We construct a symmetric matrix **L **= **D**^-1/2^**S^*'*^D**^-1/2^, where **D **is a diagonal matrix formed of the vertex degrees (), and find the eigenvectors corresponding to the *K *largest eigenvalues of **L**. Let us denote these eigenvectors by **u**_1_, **u**_2_, ..., **u**_*K*_, respectively.

6. We build a matrix **U **s.t. the *k*th column of **U **is **u**_*k *_and normalize the rows of the matrix such that each row in **U **has unit length.

7. Treating the rows of **U **as points in the *k*-dimensional Euclidean space ℝ^*K*^, we conduct a k-means clustering of these points into *K *clusters. The initial centroid positions are chosen from the data points themselves, placed as orthogonally to each other as possible.

8. We assign node *i *in the original graph to cluster *j *if and only if row *i *of **Y **was assigned to cluster *k *in the previous step. Small connected components obtained in step 4 are also merged back into the dataset in this final stage.

An important advantage of this method is that the number of clusters (*K*) can be selected automatically by evaluating the eigenvalues of **S^*'*^**. In our implementation, *K *is set to the smallest integer *k *such that *λ*_*k*_/*λ*_*k*+1 _> *ε*. *ε *is adjustable and it is chosen to be 1.02 by default. The main role of *ε *is to control the granularity of the clustering obtained: larger *ε *values tend to produce more fine-grained clusters, while a smaller *ε *yields only a few large clusters. We found that the default choice works well in a wide variety of biological problems (see the Results section). Another way to control the granularity of the clustering is to override *K *manually either before the clustering process or after the eigenvalue calculation. Both methods are facilitated by the SCPS user interface.

**Figure 2:**
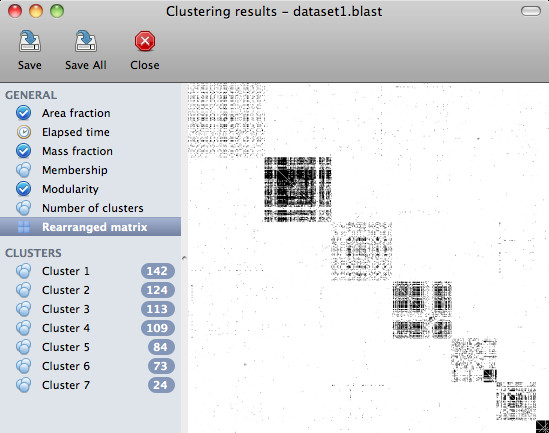
**The result viewer**. The result viewer of the graphical user interface showing the heatmap of the rearranged similarity matrix based on the calculated clustering. The individual clusters and other quality measures can be displayed by clicking on the appropriate item on the sidebar.

**Figure 3:**
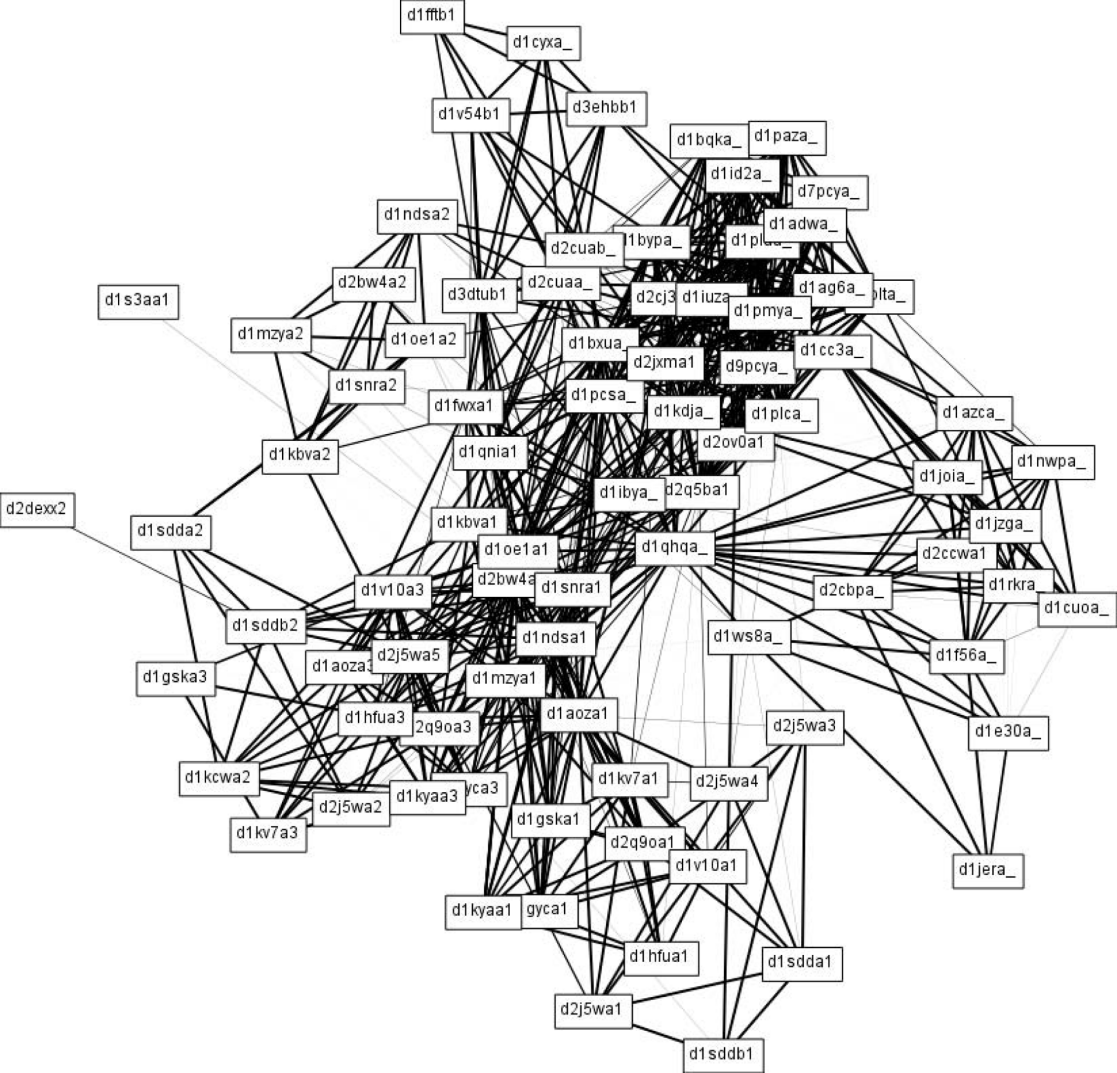
**Visualisation of a cluster calculated by SCPS**. Visualisation of a cluster as calculated and exported by SCPS.

The clustering results are presented in a separate window (see Figure 2) where the user can examine and draw the clusters one by one, calculate various quality measures (e.g., mass fraction [16] and modularity [17]), visualize the heatmap of the rearranged similarity matrix or export the results in plain text or XGMML format. XGMML files can later be loaded into Cytoscape to facilitate further visualisation and analysis. The heatmap of the rearranged similarity matrix can also be exported in publication quality to a PNG file. SCPS can also retrieve human-readable protein descriptions based on GI numbers from NCBI to aid the interpretation of the results. Clusters are drawn using the Fruchterman-Reingold layout algorithm [18], a force-directed iterative layout algorithm where nodes are considered as tiny particles that repel each other, while edges represent springs that pull the endpoints of the edge closer. The strength of the attraction force is proportional to the similarity score used in our analyses, hence the obtained layout will tend to place highly similar pairs of proteins close to each other. Figure 3 shows an example of a cluster drawing produced by SCPS.

Finally, SCPS includes a command line interface which runs the clustering without user intervention and writes the results to the standard output or to a specific output file. This enables the integration of SCPS in batch processing pipelines.

### Implementation details

SCPS uses the ARPACK library [19] for eigenvector calculations. The ARPACK library implements the implicitly restarted Arnoldi method for eigenvector calculations, which is an iterative process that is able to calculate all the eigenvectors and eigenvalues or only the top *K *ones. When one can provide a reasonable upper estimate on the number of clusters, the Arnoldi method is much more efficient than standard methods that solve the eigenvector equation directly. On the other hand, the convergence of iterative methods is affected negatively in the presence of eigenvalues with multiplicity greater than one. The multiplicity of the top eigenvalue of the affinity matrix **S **is equal to the number of connected components in the input graph. Therefore, we first eliminate small connected components of size less than five sequences from the original graph (they will not be subdivided further) and then connect the remaining components by a small amount of random edges with weight less than 0.01. This decreases the multiplicity of the top eigenvalue to one and thus improve the stability of the eigenvector calculation process without affecting the final result.

The number of clusters can be selected using one of the following methods in our implementation:

• **Automatic**. This method uses the eigengaps to select the appropriate number of clusters. *K *is set to the smallest integer *k *such that λ_*k*_/*λ*_*k*+1 _> *ε*. *ε *is adjustable and it is chosen to be 1.02 by default.

• **Bounded from above**. This method is similar to the automatic selection, but it considers at most a given number of clusters. It takes advantage of the fact that the complete eigenspectrum is not needed in this case when using the spectral clustering, saving time and resources during the computation. If the maximum number of clusters is *K*_*max*_, SCPS will compute only the top *K*_*max *_eigenvalues and the corresponding eigenvectors.

• **Exactly**. The user can select the desired number of clusters either before the analysis or after the calculation of the eigenvalues and eigengaps.

### Transforming BLAST E-values to similarities

A crucial step in the application of spectral clustering methods in the context of protein sequences is the transformation from BLAST E-values to similarities. SCPS uses an approach based on the statistical analysis of E-values within and between SCOP superfamilies. A randomly selected set of 10,000 E-values chosen from sequences within the same superfamily and 10,000 E-values chosen from sequences in different superfamilies were used to train a logistic regression model that discriminates between intra-superfamily and inter-superfamily E-values. The posterior probability returned by the model on any E-value is then interpreted as the probability of evolutionary relatedness. In case of asymmetric E-values for a pair of proteins, the lower E-value (i.e., the higher probability) is used. The proteins used for training the logistic regression model were not used later in performance assessments of the algorithm.

### Quality measures

This section describes the various quality measures we implemented in SCPS. In the following subsections, we will use the following notations:

• *s*_*ij *_is the similarity value labelling the edge between vertex *i *and *j *in a graph *G*. *s*_*ij *_= *s*_*ji *_since we always symmetrize the initial similarity values.

• *δ*_*ij *_is 1 if vertices *i *and *j *are within the same cluster, zero otherwise.

We will also need the following definitions:

**Definition 1 (Vertex weight) ***The weight of vertex i is the sum of the weight (similarity) of all its adjacent edges:d*_*i *_= Σ_*j*_*s*_*ij*_.

**Definition 2 (Cluster weight) ***The weight of cluster i is the sum of the weight of all the edges that lie fully within cluster i (i.e., both their endpoints are in cluster i)*.

#### Mass fraction

The mass fraction [16] is an internal quality measure of a clustering on a given graph *G*. Intuitively, a clustering is good if the total weight of its clusters is comparable to the total weight of the whole network; in other words, most of the heavy-weight edges are within clusters. The mass fraction simply denotes the fraction of edge weights that is concentrated inside the clusters.

**Definition 3 (Mass fraction) ***The mass fraction of a clustering defined by d*_*ij *_*is given as follows:*(1)

A disadvantage of this measure is that it attains its maximum when all the vertices are in the same cluster, hence the mass fraction alone cannot be used to decide whether a given clustering is better than another.

#### Modularity

Modularity [17] is another internal quality measure of a clustering on a given graph *G*. The idea is that it is not enough for a clustering to be good when it contains much of the edge weights within the clusters; the clustering is good when it contains *more *weight within the clusters than what we would expect if we rearranged the edges of the graph randomly while keeping the vertex weights constant. Therefore, the difference between the actual cluster weight and the expected cluster weight after such rearrangement is a good indicator of the general quality of the clustering. This measure also avoids the problem with trivial clusterings: a cluster containing all the vertices will contain exactly the same weight before and after rewiring as all the edges will stay within the same cluster, so the modularity score will be zero. Similarly, a clustering where each vertex is in its own cluster will also yield zero modularity as there are no intra-cluster edges at all.

Formally, the modularity score of a clustering is the normalized difference between the actual weight of the clusters and the expected cluster weight after a random rewiring that preserves the vertex weights. It can be shown that the expected weight of the edge between vertex *i *and *j *after rewiring is , where *m *is the sum of all edge weights in the graph (*m *= Σ_*i *≥ *j*_*s*_*ij*_) [17]. The modularity formula then follows easily:

**Definition 4 (Modularity) ***Let δ*_*ij *_*be 1 if vertices i and j are in the same cluster and zero otherwise. The modularity of the partition defined by δ is then as follows:*(2)

Positive modularity then means that there is more weight concentrated within the clusters than what we would expect from a completely random graph with the same vertex weight distribution.

#### Heatmap of the rearranged similarity matrix

This quality measure is not a single numeric value, but it provides a visual cue to the goodness of a clustering result. The basic idea is that the initial similarity matrix can be plotted as a greyscale heatmap where each pixel corresponds to a single cell of the matrix and the intensity of the pixel is proportional to the weight that the corresponding cell in the matrix represents. The rows and columns of the similarity matrix can be arranged in arbitrary order, but by arranging them in a way that rows and columns corresponding to the same cluster are next to each other, one can uncover a block-diagonal structure in the matrix if the clustering is good.

## Results and discussion

In this section, we present the results of a comparison of SCPS with other popular clustering methods (hierarchical clustering [5], connected component analysis [6] and TribeMCL [7]) on various datasets assembled from SCOP 1.75 [20], ASTRAL-95 [21] and STRING v8.1 [22]. First, we will describe the datasets we used, then we give an overview of the methods we compared SCPS with and the quality measures we used to evaluate the performance of each method. After that, the benchmark results will be presented in detail. We conclude the section with a short discussion on the scalability of SCPS.

### Data

Datasets 1-4 and the SCOP_≥ 5 _dataset in our benchmarks were taken from SCOP 1.75 [20]. Sequence data for these datasets were gathered from ASTRAL-95 [21]. Sequence data for the yeast genome benchmark were downloaded from STRING v8.1 [22] and the corresponding Gene Ontology annotations were assembled from the Saccharomyces Genome Database [23].

Datasets 1-3 are similar to the ones used in [4], but they were updated to reflect the changes in superfamily classification since the publication of the original paper. Dataset 4 was created explicitly for this study. The list of SCOP superfamilies used in each of the four datasets are listed in Table 1.

**Table 1:** List of SCOP superfamilies used in Datasets 1-4

Dataset name	SCOP superfamily ID	Size	Superfamily name
Dataset 1	46458	111	Globin-like
	47473	126	EF-hand
	49503	93	Cupredoxins
	51445	161	(Trans)glycosidases
	52833	178	Thioredoxin-like

Dataset 2	46458	111	Globin-like
	47473	126	EF-hand
	50494	99	Trypsin-like serine proteases
	51905	100	FAD/NAD(P)-binding domain
	54452	75	MHC antigen-recognition domain
	57095	76	Scorpion toxin-like

Dataset 3	46458	111	Globin-like
	47473	126	EF-hand
	51735	305	NAD(P)-binding Rossmann-fold domains
	51351	16	Triosephosphate isomerase (TIM)
	51971	9	Nucleotide-binding domain

Dataset 4	47240	66	Ferritin-like
	49899	118	Concanavalin A-like lectins/glucanases
	50494	99	Trypsin-like serine proteases
	50814	72	Lipocalins
	51905	100	FAD/NAD(P)-binding domain
	53383	92	PLP-dependent transferases
	53933	13	Microbial ribonucleases
	54236	94	Ubiquitin-like

The size of the dataset denotes the number of sequences in ASTRAL-95 corresponding to domains of the superfamily.

The SCOP_≥ 5 _dataset was constructed from SCOP 1.75 and ASTRAL-95 as follows: a database containing all sequences in ASTRAL-95 was used to conduct an all-against-all search using BLAST. For each sequence in ASTRAL-95, the corresponding superfamily was looked up from SCOP and a gold standard clustering was created using all the superfamilies that contained at least five sequences. Superfamilies containing less than five domains with associated sequence information were excluded from the benchmark, as we were interested in the performance of the methods in case of non-trivial superfamilies. The final dataset contained 632 superfamilies.

Datasets 1-4 are distributed with the downloadable SCPS package. The SCOP_≥ 5 _and the yeast genome dataset was excluded as it would have disproportionately increased the size of the package, but it is available from the authors upon request.

### Alternative clustering approaches

#### Hierarchical clustering

Hierarchical clustering is a family of clustering methods that start with individual data points (i.e. the sequences) and then build a tree by iteratively merging the closest points until only one is left [5]. The final cluster assignment is then determined by cutting the branches of the tree at a specific level. The various hierarchical clustering methods usually differ only in the way they define the distance between two sets of data points and the way they choose the optimal level to cut the branches of the tree in the end. The best results in our datasets were obtained by using the average distance metric, in which the distance between two sets of data points is given by the average distance between all possible point pairs such that one point is chosen from one of the sets and the other one is from the other set. The tree was cut at the level where the average distance metric was above 10^-6^, similarly to [4]. Pairs of proteins where BLAST did not return an E-value were considered to have an E-value of 10, which is the default BLAST E-value threshold.

#### Connected component analysis

Connected component analysis is a method that has been widely used in computer vision [6] and was initially applied to sequence clustering in GeneRAGE [2] and ProClust [3]. The method starts with a fully connected graph where the edges are labeled by the E-values or some other suitable distance metric. The algorithm proceeds by removing edges labelled by a distance larger than a given threshold, then collecting groups of vertices that still remained connected. These groups are then considered as the final result of the algorithm. The E-value threshold used in our benchmarks was 10^-6^, similarly to GeneRAGE [2].

#### TribeMCL

TribeMCL [7], a variant of the Markov clustering algorithm (MCL), models the random walk of a particle on a similarity graph, similarly to spectral clustering. A detailed comparison is given in [4], here we only note that the fundamental difference between MCL and spectral clustering is the way the random walk is propagated along the edges of the network. While our spectral clustering algorithm models the random walk exactly and analyses perturbations to the stationary distribution of the random walk, MCL modifies the random walk to promote the emergence of clusters. This approximation allows MCL to converge faster, but it can potentially lead to many small clusters. Another, less significant difference is the way TribeMCL symmetrizes the input matrix of E-values: while SCPS takes the smaller E-value in face of ambiguity and then transforms it to a similarity value, TribeMCL transforms both E-values to similarities first by taking the negative base 10 logarithm and then symmetrizes the pair by taking the average. A more detailed comparison of the two algorithms is to be found in [4].

For the TribeMCL benchmarks on Datasets 1-4, we tuned the inflation parameter of the algorithm by trying all possible values with a step size of 0.1 in the range [1.2; 5.0], as suggested by the documentation of the algorithm. The final inflation parameter was chosen in a way that resulted in the highest F-score. For the SCOP_≥ 5 _dataset, the inflation parameter was chosen as the average of the inflation parameters that were the best for Datasets 1-4.

### Comparing clusterings with a gold standard

We used the combined F-score to compare a clustering result with the gold standard SCOP superfamily classification. Let *n *denote the total number of proteins in the dataset, *n*_i* _the number of proteins in the *i*th superfamily, n_*j _the number of proteins in the *j*th calculated cluster and *n*_*ij *_the number of proteins that are in superfamily *i *and cluster *j *at the same time.

**Definition 5 (Precision) ***The precision of cluster j with respect to superfamily i is the fraction of proteins in cluster j that are also in superfamily i: p*_*ij *_= *n*_*ij *_/n_*j_

**Definition 6 (Recall) ***The recall of cluster j with respect to superfamily i is the fraction of proteins in superfamily i that are also in cluster j: r*_*ij *_= *n*_*ij*_/*n*_i*_

Now we can define the combined F-score, which combines precision and recall with equal weights.

**Definition 7 (Combined F-score) ***The combined F-score is defined as follows:*(3)

The combined F-score attains its maximum at 1 if the two clusterings are identical.

### Benchmarks on SCOP

The validity of the spectral clustering approach was tested on several datasets assembled from the SCOP database, version 1.75 [20]. Sequences were extracted from ASTRAL-95 [21], i.e. the sequence identity between any two sequences was at most 95%. Datasets 1-3 contained sequences from 5-8 protein superfamilies that were hand-chosen to resemble the datasets originally used in [4] (the original datasets could not have been re-used due to the changes in SCOP classifications and to the new sequences added to ASTRAL-95 since 2006). Dataset 4 was conceived specifically for this study. Finally, the SCOP_≥ 5 _dataset contains all the SCOP superfamilies containing at least five sequences. The datasets were described in detail earlier in the Data subsection.

The results of the spectral clustering algorithm running in fully automatic mode with default parameters (*ε *= 1.02) were compared to the results obtained from hierarchical clustering [5], connected component analysis [6] and TribeMCL [7]. The obtained partitions were compared with the gold standard SCOP superfamilies using the F-score which combines precision and recall with equal weight. The results are presented on Table 2, showing that spectral clustering clearly outperforms all the other methods at the superfamily level.

**Table 2:** Comparison of spectral clustering with other methods

	# sequences	Hierarchical clustering	CCA	TribeMCL	Spectral clustering
Dataset 1	669	0.247	0.530	0.630	**0.844**
Dataset 2	587	0.373	0.681	0.772	**0.905**
Dataset 3	567	0.253	0.588	0.625	**0.893**
Dataset 4	654	0.302	0.497	0.573	**0.685**
SCOP ≥_5_	14,183	0.393	0.530	0.576	**0.607**

The best F-score for each dataset is highlighted in bold.

**Figure 4:**
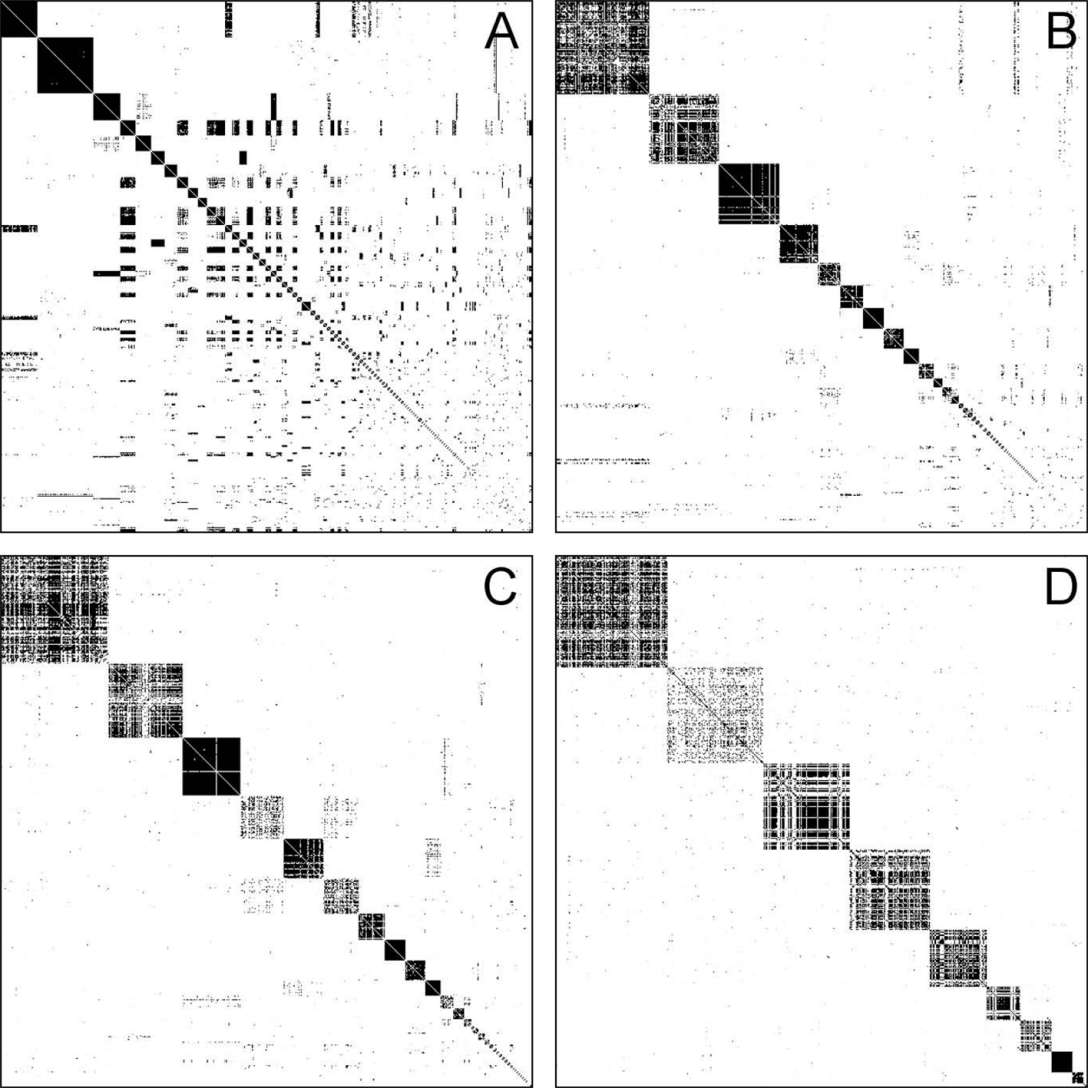
**Rearranged similarity matrices of Dataset 2 obtained from the different clustering algorithms**. Rearranged similarity matrices of Dataset 2 as obtained from hierarchical clustering (A), connected component analysis (B), TribeMCL (C) and spectral clustering (D). Rows and columns of the similarity matrices are rearranged such that sequences of the same cluster are consecutive. Each dot represents the similarity score of a sequence pair. Darker dots correspond to higher similarities.

**Figure 5:**
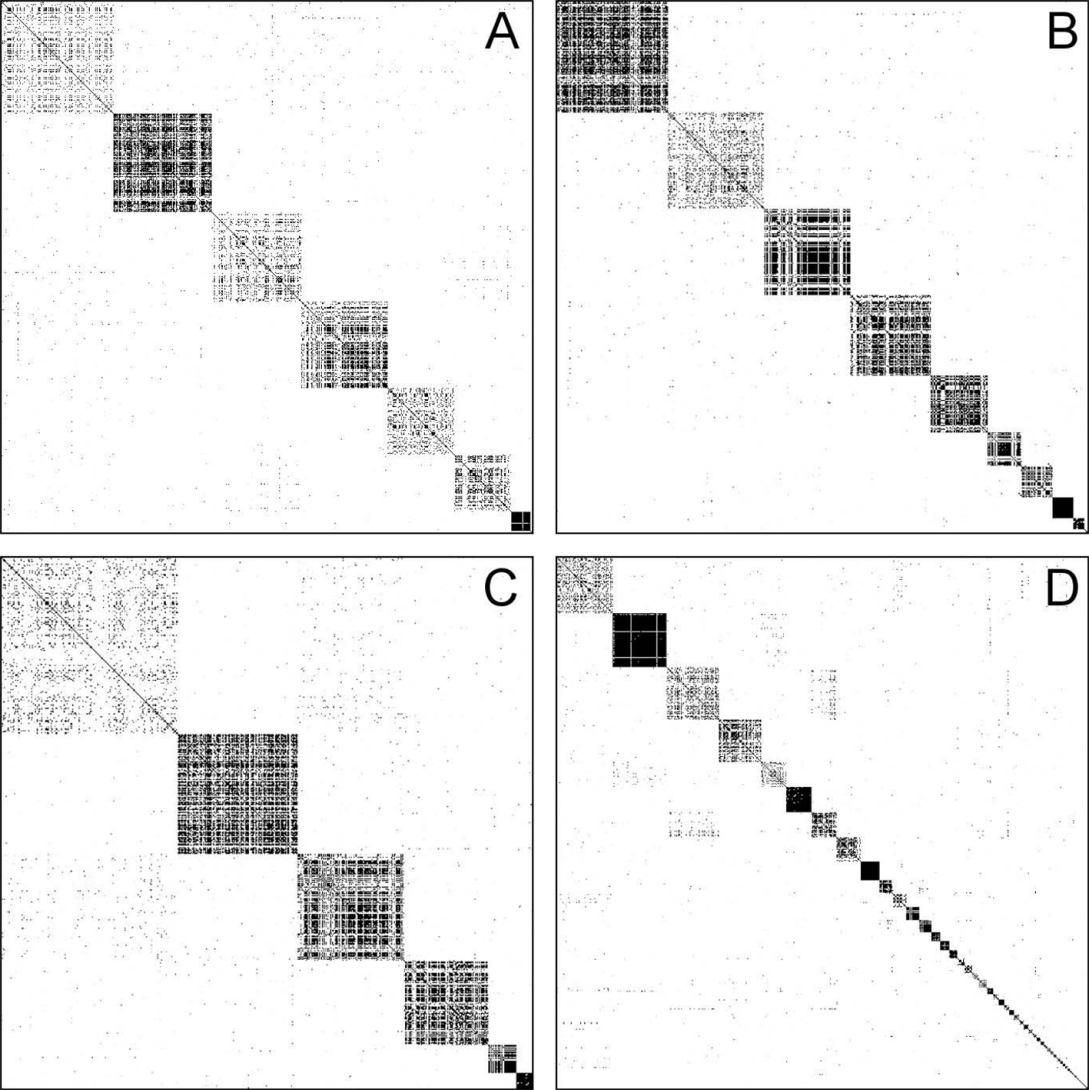
**Rearranged similarity matrices of Datasets 1-4 obtained from spectral clustering**. Rearranged similarity matrices of Datasets 1-4 as obtained from the spectral clustering algorithm. Each dot represents the similarity score of a sequence pair. Darker dots correspond to higher similarities. The block-diagonal structure of the rearranged similarity matrices confirm the good quality of the obtained clusterings.

Figure 4 compares the obtained clusterings visually using the heatmap of the similarity matrix when the matrix is rearranged such that the connections of vertices in the same cluster are placed in consecutive rows and columns. A good clustering exhibits a block-diagonal structure like the heatmaps on Figure 4C (produced by TribeMCL) and Figure 4D (produced by the spectral clustering algorithm). The hierarchical clustering (Figure 4A) and connected component analysis (Figure 4B) algorithms clearly fail to recover this structure from the input matrices. TribeMCL does a better job, but spectral clustering is able to merge some of the smaller clusters into larger compounds to improve the block-diagonality of the heatmaps. Note that all the methods perform worse on Dataset 4 than on Datasets 1-3 (see Table 2). This is due to the fact that Dataset 4 was explicitly chosen to represent a particularly difficult problem, since the superfamilies in this dataset are more divergent than those of the previous datasets, as confirmed by the heatmap visualisation of the similarity matrix (see Figure 5D).

Figure 5 shows the heatmaps of the rearranged similarity matrices for Datasets 1-4 using spectral clustering, confirming that the quality of the obtained clustering is indeed very good. It also shows us that Dataset 4 is different from the others as the number of clusters is much higher than the number of superfamilies used to construct the dataset, indicating that BLAST misses many remote homologs in this case. Using an improved similarity measure derived from PSI-BLAST [24] or CS-BLAST [25] would probably yield better results in these cases; however, examining this is out of the scope of the present paper.

### Clustering the genome of the yeast Saccharomyces cerevisiae

To further test the scalability of our method and to assess its performance on the genome of a model organism with multi-domain proteins, we collected 6,690 sequences of the yeast *Saccharomyces cerevisiae *from STRING v8.1 [22] and performed an all-against-all BLAST search on them with the default BLAST parameters. The BLAST hits were processed with spectral clustering, TribeMCL, connected component analysis and hierarchical clustering and clusters of size less than three were excluded from further assessment. The parameters for the various algorithms were the same as in the SCOP_≥ 5 _benchmark.

Owing to the lack of a hand-curated gold standard family classification for *S. cerevisiae *and the fact that proteins in a protein family tend to be functionally and structurally related, the quality of the clusters obtained were assessed by comparing them to Gene Ontology molecular function annotations [26,23]. Electronic annotations (evidence code: IEA) and annotations based on non-traceable author statements (evidence code: NAS) were ignored. For the remaining annotations and for each detected cluster of each method, multiple hypergeometric tests were performed to assess the statistical significance of the occurrence of GO molecular function terms within the cluster at a significance level of 0.05. Correction for multiple hypothesis testing was performed by controlling the false discovery rate (FDR) using the Benjamini-Hochberg method [27]. We treated a cluster as significant if at least one of the GO terms were overrepresented within the cluster and then calculated the total number of significant clusters divided by the total number of clusters containing at least three proteins, as well as the total size of significant clusters divided by the total size of clusters containing at least three proteins. These ratios along with the exact numbers are reported in Table 3. Although hierarchical clustering and connected component analysis achieve the highest significant cluster ratio (0.829 and 0.792, respectively) when taking into account the cluster counts only, they both generate a high number of singletons and clusters of size two, therefore the significant clusters cover only a small fraction of the whole similarity graph (1,166 and 1,858 sequences out of 6,690). The difference becomes clear when the same ratios are calculated using the cluster sizes instead of the cluster counts: spectral clustering dominates with 4,863 sequences in 235 significant clusters covering 90.3% of the 5,380 sequences that are in clusters of at least size three, while TribeMCL comes second with 3,600 sequences in 245 clusters covering 88.9% of the 4,047 sequences in clusters of size ≥ 3. One may argue that this difference can be attributed to the fact that spectral clustering tends to produce fewer and thus larger clusters than MCL, but the same difference between spectral clustering and TribeMCL can also be seen in the unweighted (cluster counting) case (76.3% versus 73.8%). Similar results were obtained when we used the MIPS FunCat annotations [28] instead of the Gene Ontology (data not shown). These results illustrate that spectral clustering is a viable alternative to other popular methods even on a genome-wide scale.

**Table 3:** Comparison of the results obtained on the genome of the yeast Saccharomyces cerevisiae

		Hierarchical clustering	CCA	TribeMCL	Spectral clustering
**Cluster count**	Significant	243	243	245	235
	All	293	307	332	308
	Ratio	0.829	0.792	0.738	0.763

**Total cluster size**	Significant	1,166	1,858	3,600	4,863
	All	1,396	2,144	4,047	5,380
	Ratio	0.835	0.866	0.889	0.903

### Scalability considerations

The spectral clustering method has two potential bottlenecks. One of them is the k-means clustering step where no exact result is known about the number of steps the algorithm takes in the worst case. However, it was shown recently that the k-means clustering procedure terminates in a polynomial number of steps with high probability in high-dimensional spaces when the data points are drawn from independent multivariate normal distributions [29]. It was also proven that given a clustered structure in the original input dataset, data points of the same cluster will be aligned roughly along orthogonal directions in our k-means step. The normalisation step then ensures that these points will be situated close to each other [15], thus they can be approximated well with multivariate normal distributions. Therefore, the data points we are likely to encounter in the k-means step satisfy the conditions of polynomial time complexity. The other potential bottleneck of the algorithm is the calculation of the eigenvectors. Typically, the number of steps required to calculate the top *K *eigenvectors scales linearly with the number of non-zero elements in the input matrix when using the implicitly restarted Arnoldi method [19]. Since SCPS uses this method when a maximum cluster count is specified, the algorithm is expected to terminate in polynomial time for real sequence similarity datasets, enabling us to analyse large datasets comprising of thousands of protein sequences. In our experiments, the SCOP_≥ 5 _dataset was processed in 83 minutes using a single core of a quad-core Intel Xeon X3360 desktop machine running at 2.83 GHz, using the top 2000 eigenvalues and eigenvectors of the similarity matrix. This does not include the CPU time required to run the all-against-all BLAST query on SCOP, which took nearly four hours.

## Conclusions

In this paper, we presented SCPS, an efficient, user-friendly, scalable and platform-independent improved implementation of a spectral clustering method [4], which can identify protein superfamilies in datasets containing thousands of proteins within a few minutes. The software along with its source code is available to non-commercial users free of charge. We would like to encourage users and developers to provide feedback, suggest new features or contribute code. Future work will focus on the improvement of the similarity measure used by the algorithm and a parallelized implementation of the method to exploit the power of multiple CPU cores.

## Availability and requirements

Project name: SCPS

Project home page: http://www.paccanarolab.org/software/scps

Operating systems: Windows, Mac OS X, Linux

Programming language: C++

License: GNU General Public License (GPL) v3

Restrictions to use by non-academics: None

## Authors' contributions

TN designed and implemented the SCPS graphical and command line interfaces, contributed robustness, stability and scalability improvements to the original algorithm and performed benchmarks. RS provided important biological insights and ideas to the algorithm and the datasets and extensively tested the software. AP conceived and implemented the first version of the algorithm, contributed valuable ideas to the present implementation and tested the algorithm. TN, RS and AP contributed to the writing of the paper. All the authors have read and approved the final manuscript.
